# Cross-cultural validation of simplified Chinese version of spine functional index

**DOI:** 10.1186/s12955-017-0785-7

**Published:** 2017-10-18

**Authors:** Xiao-Yi Zhou, Xi-Ming Xu, Jian-Ping Fan, Fei Wang, Sui-Yi Wu, Zi-Cheng Zhang, Yi-Lin Yang, Ming Li, Xian-Zhao Wei

**Affiliations:** 1 Department of Orthopaedic Surgery, Changhai Hospital, Second Military Medical University, 168 Changhai Road, Shanghai, 200433 China; 2 Department of Spinal Surgery, Changzheng Hospital, Second Military Medical University, Shanghai, China; 3 Faculty of Naval Medicine, Second Military Medical University, Shanghai, China

**Keywords:** Spinal functional index, Musculoskeletal disorders, Confirmatory factor analysis, Structural validity

## Abstract

**Background:**

No effective constructs were available in mainland China to assess the whole spine function. The SFI was developed to evaluate spinal function based on the concept of a single kinetic chain concept for whole spine. The SFI has been translated to Spanish and Turkish with accepted psychometric properties. It is imperative to introduce the SFI in mainland China and further to explore the measurement properties.

**Methods:**

The English versions of the SFI was cross-culturally translated according to international guidelines. Measurement properties (content validity, construct validity and reliability) were tested in accordance with the COSMIN checklists. A total of 271 patients were included in this study, and 61 participants with neck pain and 64 participants with back pain paid a second visit three to seven days later. Confirmatory factor analysis (CFA) and principal factor analysis (PCA) were applied to test the factor structure. The Functional Rating Index (FRI), Neck Disability Index (NDI), Oswestry Disability Index (ODI), SF-12 and a Visual Analogue Scale (VAS) were employed to evaluate the construct validity. Cronbach’s alpha and an intra-class correlation coefficient (ICC) were calculated for internal consistency and reproducibility.

**Results:**

The means score of SC-SFI was 63.60 in patients with spinal musculoskeletal disorders. A high response rate was acquired (265/271). No item was removed due to abnormal distribution or low item-total correlation. Results of CFA did not support that one-factor structure was in goodness of fit (CMIN/DF = 3.306, NNFI = 0.687, CFI = 0.756, GFI = 0.771 and RMSEA = 0.092). Yet, PCA suggested a one-factor structure was the best, accounting for 32% of the total variance. For structural validity, the SC-SFI correlated highly with the FRI, NDI, ODI, and PF, BP in SF-12 (*r* = 0.661, 0.610, 0.750, 0.709, 0.605, respectively). All the a priori hypotheses were verified. The Cronbach’s alpha for the SC-SFI was 0.91, and ICC was 0.96 (95% CI, 0.94–0.98). Bland-Altman plot also confirmed excellent test-retest reliability.

**Conclusions:**

The SFI has been culturally adapted into SC-SFI with remarkable clinical acceptance, excellent internal consistency, reproducibility, and construct validity when applied to patients with spinal musculoskeletal disorders. The results of current study suggest that SC-SFI can be applied by physicians and researchers to measure whole-spine functional status in mainland China.

**Electronic supplementary material:**

The online version of this article (10.1186/s12955-017-0785-7) contains supplementary material, which is available to authorized users.

## Background

Spinal musculoskeletal disorders are becoming a growing concern globally due to the high morbidity and economic loss, affecting almost 10% population worldwide and 20% population in China [5, 22]. They are the common causes of severe long-term pain and physical disability, making patients absent from work and deteriorating their quality of life [13, 15]. Patient reported outcome (PRO) measures are increasingly being recommended for use in clinical practice to assess the patients’ pain level, function limitation, quality of life and health status [19]. International consensus on a standard set of outcome domains accompanied PROs and contributing factors is needed to assess patients with spinal disease [9]. Quantification of patients’ subjective changes could assist physicians, surgeons and therapists to evaluate the function and symptoms as well as the intervention outcomes. Thus, it is imperative to introduce and adopt these PRO measures into clinical practices.

Multiple PRO measures have been developed to evaluate musculoskeletal problems. Yet, most of the studies concentrated on the neck or back problems respectively. They did not recognize the spine as a whole unit [3]. Commonly used back pain and function measures include Oswestry Disability Index (ODI), the Quebec Back Pain Disability Scale (QDS) and the Roland Morris Disability Questionnaire (RMDQ) [8, 11, 14]. The Neck Disability Index (NDI) and the neck pain and disability scale (NPDS) are the most widely used tool to measure functional restrictions due to neck pain [25, 28]. Currently, whole spine specific PRO instruments includes Functional Rating Index (FRI) and Core Outcomes Measures Index (COMI) [10, 6]. A simplified Chinese version of FRI has been translated with validation across cultures. For patients with multiple area spinal pain, the FRI is provisionally recommended for the evaluation of disability because of its positive results for internal consistency, structural validity, hypothesis testing and responsiveness [16]. The other tool – COMI - has not yet been introduced in mainland China. Nevertheless, both measures are restricted for whole spine function evaluation with limitation in validation or administration [27].

Spine Function Index (SFI), a patient-reported outcomes designed for the single kinetic chain concept, was proposed in order to assess the spinal function as a whole unit [11].

The SFI has been cross-culturally adapted into Spanish [4] and Turkish [24] with good psychometric properties. Currently, no validated simplified Chinese version of SFI (SC-SFI) has been published. Thus, it is important to adapt the SFI into a simplified Chinese version.

The purposes of present study were: 1) to translate and cross culturally adapt the English version of SFI into simplified Chinese; 2) to test the measurement properties of SC-SFI according to COSMIN checklist in mainland China.

## Methods

### Translation and cross-cultural adaptation

The translation and cross-cultural adaptation of the SFI English version to simplified Chinese version was performed using a forward and backward method [12]. The forward translation was performed independently by two bilingual translators whose mother tongue was Mandarin Chinese. One translator, the author of this article (X-YZ), was aware of the purpose of the translation and concepts of the questionnaires. The other translator was an English professor with no medical background as well as research aims. After comparing the two translation versions of the SC-SFI, discrepancies were discussed and reconciled by consensus. The back translation was performed blindly by two independent native English speakers, who lacked medical background. Each English translation was then compared with the original English SFI Questionnaire and checked for inconsistencies by the committee. Consensus was reached on the semantic, idiomatic, and conceptual equivalence between the original English edition and the SC-SFI edition. Finally, the SC-SFI Questionnaire was pilot tested in a cohort of 25 patients with spinal musculoskeletal disorders. Each patient completed the SC-SFI Questionnaire and was asked for difficulties in filling out the questionnaire or understanding the aim and meaning of each question. The committee discussed all the findings and then established the final version of SC-SFI Questionnaire.

### Participants

A total of 271 patients with a diagnosis of a spinal musculoskeletal disorders were recruited from the outpatient department of orthopedics in Changhai hospital of the Second Military Medical University between July 2014 and March 2015. The inclusion criteria were: age over 18 years old, ability to read and write Chinese, symptoms duration for 12 weeks or more, and being diagnosed by a medical practitioner with a diagnosis of a musculoskeletal spine condition or symptoms. Patients were excluded from the study if the diagnosis were: tumors, infection, pregnancy, systemic rheumatologic disease, ankylosing spondylitis, late-stage surgery, neurological diseases and psychiatric diseases. In addition, a total of 61 patients with neck disorders and 64 patients with lumbar disorders were asked to paid a second time to filling out the questionnaires three to seven days later. This study was approved by the Human Research Ethics Committee of the Second Military Medical University, and written informed consent were obtained from each participant.

### Instruments

#### Spine functional index (SFI)

The SFI consists of 25-items with a three-point response option of ‘Yes’, ‘Partly’ and ‘No’. The score is calculated by summing the 25-item then multiplied by four to provide a percentage scale and subtracted from 100 to generate score associative with the patients’ functional status. Up to two missing responses are permitted.

#### Functional rating index (FRI)

The original FRI contains 10 items scoring from 0 to 4 in regard to the physical functional status. The final score was calculated by summing up the item scores, then dividing by the possible total points and multiplying by 100%. The scores ranged from 0% (no pain or disability) to 100 (worst pain or disability). One missing response is allowed [16].

#### Owestry disability index (ODI)

The measurement of ODI contains 10 items scoring from 0 to 5 in regarding to low back status. The ODI score is calculated by doubling the summation of 10 items and is considered as a percentage of the patient’s subjective disability [18].

#### Neck disability index (NDI)

The instrument of NDI is an alteration of ODI, containing 10 items scoring from 0 (no activity limitations) to 5 (major activity limitations). The NDI score is converted to a percentage by doubling the sum of 10 items and can be used to assess disability [29].

#### Short form 12 (SF-12)

The SF-12 questionnaire is a self-administered instrument derived from SF-36, organized into eight domains: physical functioning (PF), role limitations due to physical health problems (RP), bodily pain (BP), general health (GH), vitality (VT), social functioning (SF), role-emotional (RE), and mental health (MH). The raw scale scores are linearly transformed to a 100-point scale. A Chinese version of SF-12 has already been adapted and widely used to measure generic health status [17].

#### Visual analogue scale (VAS)

The VAS questionnaire is a 100 mm horizontal line with “no pain” written at the left end point and “worst pain” written at the right end point .

All participants were required to complete four questionnaires, which consisted of the SC-SFI, FRI, SF-12 and VAS. The ODI or NDI questionnaire was filled out if the participant complained neck pain or low back pain, respectively.

#### Statistical analysis

Measurement properties (content validity, construct validity, and reliability) were analyzed according to the COSMIN checklist [20, 21].

### Content validity

To cross-culturally adapt the SFI into a simplified Chinese version, all the items need to be analyzed. Items with score distribution out of normal range (Z-skewedness value more than 1.96) or have poor relationships with other items (item-total correlation coefficient less than 0.30) should be excluded in the SC-SFI [26].

### Construct validity

Construct validity is to describe whether the construct could measure the concept, which includes structural validity, hypothesis testing, and cross-cultural validity [18]. Structural validity is meant to explore the underlying structure of the SC-SFI, and confirmatory factor analysis (CFA) is preferred for cross-cultural studies. Hypotheses are proposed based on the conceptual relationships between the questionnaires [7].

### Structural validity

The study used CFA to test whether the one-factor structure was suitable in the Chinese version, which was proposed by Tonga et al. [24]. A best-fit model should present a non-significant chi-square result and the following indices: (1) a Satorra–Bentler scaled chi-square (S-Bχ ^2^)/degrees of freedom ratio (CMIN/DF) of 2.0 or less; (2) a non-normed fit index (NNFI) no less than 0.90; (3) a Robust-Comparative fit index (Robust-CFI) no less than 0.90; (4) a goodness-of-fit index (GFI) no less than 0.90; and (5) a low root mean square error of approximation (RMSEA) no less than 0.08 [2]. Considering that the SFI has only applied in three countries and no stable factor structure was proposed, principal component analysis (PCA) was preferred to explore the structure of the SC-SFI. An eigenvalue over 1 and item loading over 10% were used to determine the factor number.

#### A priori hypotheses

The purpose of the SFI was to assess the function of whole spine, which results should be correlated highly with the FRI, NDI and ODI, which also were used to assess the function of cervical or lumbar spine. Also, the SC-SFI should correlate highly with PF in SF due to both were designed to evaluation pain related functional restriction. Because MH in the SF-12 was designed to measure the mental health, which correlated low with pain related functional restriction, thus, the SC-SFI should correlate moderately with MH in the SF-12. Therefore, priori hypotheses were proposed as following:

1. The SC-SFI should correlate highly with the FRI;
2. The SC-SFI should correlate highly with the ODI;
3. The SC-FRI should correlate highly with the NDI;
4. The SC-PCS should correlate highly with PF in the SF-12;
5. The SC-PCS should correlate low with MH in the SF-12;
6. The SC-PCS should correlate moderately with the VAS;

The correlation values were classified as follows: low: *r* = 0.00–0.30; moderate: *r* = 0.31–0.60; high: *r* ≥ 0.60. *P*-values <0.05 were considered to indicate statistical significance [1].

### Internal consistency and test-retest reliability

Cronbach’s α was calculated to explore the internal consistency of the SC-SFI. Excellent was deemed when Cronbach’s α was between 0.80 and 0.95.

Blant-Altman plot and intra-class correlation coefficient (ICC) were used to assess the test-retest reliability. An ICC value over 0.70 was deemed as excellent reliability [23].

Statistical Package for the Social Sciences (SPSS) version 18.0 (IBM, Armonk, NY, USA) was applied to proceed statistical analysis. AMOS 18.0 (Chicago, Illinois) was used to perform CFA. Numerical data are expressed as the mean values ± the standard deviation (SD). *P* value of less than 0.05 was considered statistically significant.

## Results

### Cross-cultural translation and adaptation

The SFI was successfully translated into simplified Chinese. Nearly all the participants could finish the questionnaire with ease. Still, some cross-cultural modification were made: item 2 “I change position frequently for comfort” was translated as “因不舒服频繁变换姿势” rather than “为了舒服点经常变换姿势”; “5 kg or 10 lbs” was translated as “5公斤” because lbs. was not commonly used in daily life in China (Additional file 1).

### Patient characteristics

A total of 271 participants were recuited and completed the investigation with a response rate of 98% (265/271). Detailed description was presented in Table 1. There were 118 patients with cervical disorders, 142 patients with lumbar disorders and 11 patients with both regional disorders. The mean SC-SFI score was 63.60 and the gross mean pain duration was 17 months.

**Table 1:** Demographic characteristics of patients

	Mean/N	SD
Male/Female	116/155	
Subregion		
Cervical	118	
Lumbar	142	
Multi	11	
FRI	36.72	18.75
VAS	48.22	21.03
NDI	27.75	11.88
ODI	30.17	15.25
SFI	63.60	20.09
SF-12		
PF	75.20	19.71
RP	45.66	37.66
BP	54.93	17.13
GH	55.24	18.11
VT	64.17	18.31
SF	72.42	17.82
RE	56.33	39.85
MH	66.61	16.02

*SD* standard deviation, *N* number, *FRI* functional rating index, *VAS* visual analogue scale, *ODI* Oswestry disability index, *NDI* neck disability index, *SFI* spinal function index, *GH* general health perception, *PF* physical functioning, *RP* role limitations due to physical health, *RE* role limitations due to emotional problems, *BP* bodily pain, *MH* mental health, *VT* vitality, *SF* social functioning

### Content validity

Response trend analysis found no item was scored out of normal distribution with skewedness over 1.96. Also, none of the items was correlated with the total items less than 0.30. Therefore, all the 25 items were included in the SC-SFI (Table 2).

**Table 2:** Item-deleted Cronbach’s alpha, corrected item-total correlation and response trend for each item in the SC-SFI

	Item-deleted Cronbach’s Alpha	Corrected item-total correlation	Z-skewness
Item 1	0.91	0.45	0.19
Item 2	0.91	0.41	0.14
Item 3	0.90	0.65	0.92
Item 4	0.91	0.42	0.53
Item 5	0.90	0.58	0.24
Item 6	0.91	0.33	0.07
Item 7	0.91	0.47	1.02
Item 8	0.91	0.33	−0.76
Item 9	0.90	0.51	0.36
Item 10	0.90	0.69	0.52
Item 11	0.90	0.66	0.61
Item 12	0.91	0.31	0.17
Item 13	0.90	0.51	1.58
Item 14	0.90	0.54	0.68
Item 15	0.91	0.44	0.57
Item 16	0.91	0.38	0.07
Item 17	0.90	0.48	0.97
Item 18	0.91	0.43	1.60
Item 19	0.91	0.47	1.17
Item 20	0.90	0.58	0.06
Item 21	0.90	0.61	0.63
Item 22	0.90	0.66	0.84
Item 23	0.90	0.62	0.68
Item 24	0.90	0.51	0.31
Item 25	0.90	0.56	0.98

### Missing items

Nearly all the items were appropriated fully responded. Items 9, 14 and 22 were missed two times, and item 19 was missed once.

### Construct validity

#### Structural validity

**Fig. 1:**
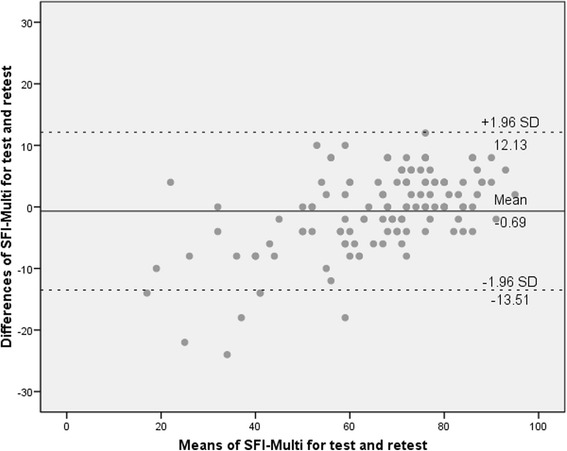
The screen plot of the eigenvalues against the component numbers for SC-SFI

CFA was performed to assess whether one-factor structure was suitable in the SC-SFI. Results showed that none of the parameters supported an excellent structure simulation after adjustment (see Fig. 1). The CMIN/DF was 3.306, NNFI was 0.687, CFI was 0.756, GFI was 0.771 and RMSEA was 0.092.

The results of PCA suggested a one-factor structure were the best, accounting for 32% of the total variance. In the subgroups of patients with neck pain or back pain, PCA also suggested a one-factor structure with 31% or 28% variance included (see Table 3). Scree plot were performed and presented one-factor structure were suitable among the total and subgroup participants.

**Table 3:** Adjusted principle component analysis for the SC-SFI

	Item distracted indices			Item loading		
	Total (*n* = 271)	Neck pain (*n* = 118)	Back pain (*n* = 142)	Total (n = 271)	Neck pain (n = 118)	Back pain (n = 142)
Item 1	0.61	0.74	0.61	0.49	0.53	0.48
Item 2	0.52	0.61	0.71	0.44	0.13	0.32
Item 3	0.59	0.76	0.67	0.69	0.57	0.60
Item 4	0.55	0.64	0.68	0.46	0.47	0.33
Item 5	0.67	0.67	0.65	0.64	0.67	0.35
Item 6	0.69	0.40	0.66	0.36	0.36	0.45
Item 7	0.57	0.74	0.76	0.52	0.49	0.55
Item 8	0.64	0.74	0.69	0.35	0.20	0.34
Item 9	0.73	0.76	0.56	0.57	0.77	0.70
Item 10	0.60	0.75	0.65	0.75	0.77	0.67
Item 11	0.65	0.68	0.59	0.71	0.39	0.48
Item 12	0.56	0.50	0.59	0.34	0.67	0.51
Item 13	0.53	0.58	0.64	0.56	0.73	0.49
Item 14	0.55	0.73	0.76	0.59	0.55	0.55
Item 15	0.59	0.71	0.60	0.47	0.39	0.49
Item 16	0.71	0.59	0.57	0.41	0.50	0.47
Item 17	0.57	0.65	0.66	0.54	0.47	0.57
Item 18	0.59	0.71	0.61	0.48	0.53	0.44
Item 19	0.53	0.70	0.66	0.52	0.44	0.54
Item 20	0.59	0.56	0.66	0.65	0.64	0.57
Item 21	0.73	0.79	0.66	0.69	0.72	0.63
Item 22	0.61	0.65	0.65	0.71	0.74	0.67
Item 23	0.74	0.70	0.73	0.69	0.64	0.70
Item 24	0.76	0.77	0.73	0.59	0.53	0.54
Item 25	0.51	0.69	0.55	0.62	0.44	0.67

#### A priori hypotheses

The SC-SFI correlated highly with the FRI, NDI, ODI, and PF, BP in SF-12. Moderate correlation was found between the SC-SFI and VAS. Low correlation was found between the SC-SFI and MH in SF-12. Specifically, the SC-SFI correlated highly with the NDI in neck pain patients and highly with the ODI in patients with back pain. Thus, all the priori hypotheses were verified (Table 4).

**Table 4:** Correlation between SC-SFI and spinal function related measures

	VAS	FRI	NDI	ODI	PF	RP	BP	GH	VT	SF	RE	MH
SFI (Total *n* = 261)	−.481^**^	−.661^**^	−.610^**^	−.0.750^**^	.709^**^	.605^**^	.640^**^	.316^**^	.346^**^	.549^**^	.387^**^	.286^**^
SFI (Neck pain *n* = 118)	−.628**	−.448**	−.694**		.612**	.617**	.631**	.274	.310**	.530**	.325**	.348**
SFI (Back pain *n* = 142)	−.612^**^	−.423^**^		−.770^**^	0.539**	.549^**^	.629^**^	.397^**^	.489^**^	.594^**^	.466^**^	.384^**^

**:moderately correlated

### Internal consistency and test-retest reliability

**Fig. 2:**
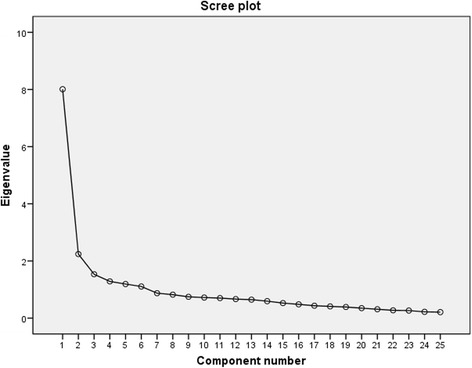
The Bland-Altman plot for test-retest agreement of SC-SF

The SC-SFI presented excellent internal consistency, with Cronbach’s alpha values were 0.91, 0.90 and 0.89 in the total, neck pain and back pain patients, respectively. The ICCs for the SC-SFI were 0.96 (95% CI, 0.94–0.98), 0.94 (95% CI, 0.91–0.97) and 0.96 (95% CI, 0.95–0.97) in total, neck pain and back pain patients. Bland-Altman plots also demonstrated that no significant differences were between the measures from the two test sessions (see Fig. 2).

## Discussion

In this study, the SFI was successfully translated into simplified Chinese with excellent construct validity and reliability in mainland China. Notably, the SC-SFI was easy to administrate with high completion rate and low missed responses.

After cultural adaptation, the Chinese version of SFI was readable and apprehensible for patients with spinal musculoskeletal disorders in mainland China. The SC-SFI had outstanding clinical acceptability with high completion rate. In Gabel et al.’s study, they found there might be redundant items in the scale [12]. Thus, we assess the content validity of the SC-SFI to exclude items that were not measuring the same concepts with the other items. Both response trends and item-total correlations revealed no items were ought to be removed. Therefore, the SC-SFI consisted 25 items the same as the original questionnaire. Gabel et al. found it took 2 min to complete the questionnaire, which was acceptable during clinical practices [12]. In our study, it was impractical to record the filing out time of the SC-SFI because all the participants were asked to finished several questionnaires at a time. So did the Turkish and Spanish studies [4, 24].

CFA was recommended to investigate the factor structure in cross-cultural studies according to the COSMIN studies. Considering the conceptual foundation and results of the English, Turkish and Spanish studies, one-factor structure was suitable for the SFI. However, we found one-factor structure was not in perfect goodness of fit for the SC-SFI after CFA, indicating there might be more complex structure underling SFI. Then, we performed PCA to explore the best structure. Based the results on eigenvalues, variance loading and scree plot, one-factor structure was appropriate for the SC-SFI, just the same as the English, Turkish and Spanish studies. To be noticed, item 8, 12 and 16 had comparable low item loading, which could decrease the efficacy of the structure. PCA produced one-factor structure while CFA could not verify one-factor structure, indicating there were underling implicit structure. These results also indicated removal some items could increase both the variance loading and goodness of fit, thereby to produce a solid factor structure.

Construct validity was defined to assess the extent to which a test measures what it claims, or purports, to be measuring. In agreement with the recent published COSMIN studies, a priori hypotheses should be proposed before carrying out the project. And a construct has good construct validity when 75% of the hypotheses are confirmed. In our study, all the hypotheses were confirmed as evidenced by correlations between the SC-SFI and other related measures. The SFI was designed to assess the whole function of spine just as the FRI. The NDI questionnaire was commonly used to evaluate neck pain while the ODI was accepted tool for lumbar disorders. Therefore, the SFI should had high correlations with the FRI, NDI and ODI, which were demonstrated by multiple correlations. Gabel et al. also found that the SFI had a high correlation with FRI (*r* = 0.85) while Tonga found that the SFI had moderate correlation with FRI in Turkish participants. In our study, the SC-SFI had high correlation with NDI (*r* = 0.61) and ODI (*r* = 0.75), which results were similar to Tonga’s findings (SFI vs. NDI and ODI: *r* = 0.58, *r* = 0.72) [24]. In the Spanish study, the SFI was found to had moderate correlation with NDI (*r* = 0.46) [4]. Aggregating all the findings, we could conclude that the SC-SFI demonstrated with excellent construct validity in assessing patients with spinal musculoskeletal disorders.

The SC-SFI showed excellent internal consistency with Cronbach α value of 0.96, indicating all the items were intended to assess spinal function. The Cronbach α found in our study is also in line with those reported in English (α = 0.91), Turkish (α = 0.85) and Spanish (α = 0.85) studies [4, 12, 24], indicating that SFI remained internal stable across cultures. Both ICC analysis and Bland-Altman plot proved SC-SFI had exceptional test-retest reliability, indicating that SC-SFI was capable of assessing functional status over time. Other versions of SFI also demonstrated excellent reproducibility (English, ICC = 0.97; Turkish, ICC = 0.93; Spanish, ICC = 0.96). Therefore, the SFI remained stable across cultures.

Although the findings of present study provide strong support for validation of SC-SFI, a few limitations should be noticed. First, the SC-SFI were not applicable to entire Chinese-speaking population. Because traditional Chinese characters were commonly used in regions like Taiwan, Hong Kong and other Chinese communities worldwide where Cantonese was speaking. Second, this study only included participants from outpatient clinics. Further studies should be performed in inpatient or community settings. Third, responsiveness was not tested in this study, which required long-term follow-up. We would proceed this task in future studies.

## Conclusions

The SFI has been culturally adapted into simplified Chinese with remarkable clinical acceptance, excellent internal consistency, reproducibility, and construct validity when applied to patients with spinal musculoskeletal disorders. The results of the current study suggest that the SC-SFI can be applied by physicians, researchers and rehabilitation providers to measure whole-spine functional status in mainland China.

## Additional file

Additional file 1: Simplified Chinese version of SFI. (DOCX 27 kb)

## Abbreviations

BP: bodily pain
CFA: confirmatory factor analysis
CMIN/DF: degrees of freedom ratio
COMI: Core Outcomes Measures Index
FRI: Functional Rating Index
GFI: goodness-of-fit index
GH: general health
ICC: intra-class correlation coefficient
MH: mental health
NDI: Neck Disability Index
NNFI: non-normed fit index
NPDS: neck pain and disability scale
ODI: Oswestry Disability Index
PCA: principal component analysis
PF: physical functioning
PRO: Patient reported outcome
QDS: Quebec Back Pain Disability Scale
RE: role-emotional
RMDQ: Roland Morris Disability Questionnaire
RMSEA: root mean square error of approximation
Robust-CFI: Robust-Comparative fit index
RP: role limitations due to physical health problems
S-Bχ ^2^: Satorra–Bentler scaled chi-square
SC-SFI: simplified Chinese version of Spine Function Index
SF: social functioning
SF-12: Short form 12
SFI: Spine Function Index
SPSS: Statistical Package for the Social Sciences
VAS: Visual Analogue Scale
VT: vitality
