# Supplementary material for: Cross-cultural validation of simplified Chinese version of spine functional index
Source: Health Qual Life Outcomes. 2017 Oct 18;15:203. doi: 10.1186/s12955-017-0785-7 (PMC5648461; doi:10.1186/s12955-017-0785-7)
Supplement: Additional file 1: — Simplified Chinese version of SFI. (DOCX 27 kb) [file 12955_2017_785_MOESM1_ESM.docx]

**脊柱功能指数（SFI） 日期：**

**姓名： 疼痛 □颈部 □中背 □下腰部**

请完成以下内容：

脊柱（背部和/或颈部）不适给日常活动带来困难。这项列表包含了人们在有这样问题时经常描述的语句。回忆一下自身现在或者近几日情况，在描述到的条目上做标记；如果没有描述到，保留空格；如果一个条目部分描述，做半个（1/2）标记。

**依据自身脊柱填写**

无 部分 有

**SFI得分：计算上述部分—标记的方框**

**总分（SFI得分） 100分：100 --（总分×4）= %**

**MDC(90%可信度)：颈部**=6.9.%或1.7SFI得分;  **中下背**=5.9%或 1.5SFI得分。

**全脊柱**=6.5%或1.6SFI得分。低于此值可能有错误。

- □ □ 17.穿衣需要帮助或者动作缓慢。
- □ □ 18.在床上活动困难。
- □ □ 19.有困难来集中精力和/或读书。
- □ □ 20.影响坐立。
- □ □ 21.坐下和起立困难。
- □ □ 22.仅能站立较短的时间
- □ □ 23.蹲下和/或跪下困难。
- □ □ 24.弯腰困难。（例如：捡东西，穿袜子）
- □ □ 25.爬楼梯缓慢或用扶手。
- □ □ 9.步行、正常娱乐、运动活动受到影响。
- □ □ 10.做日常家务有困难。
- □ □ 11.睡眠不佳。
- □ □ 12.需要个人护理帮助。例如：洗衣服，个人卫生等。
- □ □ 13.平时的日常活动（工作、社交）受到影响。
- □ □ 14.变得易怒和/或坏脾气。
- □ □ 15.变得更虚弱和/或僵硬。
- □ □ 16.自主出行受到影响（开车、公共交通工具）。
- □ □ 1.大部分时间待在家里。
- □ □ 2. 因不舒服频繁变换姿势。
- □ □ 3.不能干重活（拖地、举起5公斤重物、园艺劳动）。
- □ □ 4.经常休息。
- □ □ 5.要求他人替我做事情。
- □ □ 6.总有疼痛或其他不适。
- □ □ 7.举起或者搬运困难（例如:包裹，购物满5公斤）。
- □ □ 8.食欲不佳。
